# Exploring preferences of different modes of administration of hypomethylating agent treatments among patients with acute myeloid leukemia

**DOI:** 10.3389/fonc.2023.1160966

**Published:** 2023-05-08

**Authors:** Audrey Delmas, Laurie Batchelder, Ira Arora, Solene Bayet, David Bruhn, Alice Eberhardt, Stephanie Philpott, Ana Maria Rodriguez-Leboeuf

**Affiliations:** ^1^ Otsuka Pharmaceutical Europe Ltd., Wexham, United Kingdom; ^2^ IQVIA, Reading, United Kingdom; ^3^ IQVIA, London, United Kingdom; ^4^ IQVIA, Courbevoie, France; ^5^ Otsuka Pharmaceutical Development & Commercialization Inc., Rockville, MD, United States; ^6^ Otsuka Pharma GmbH, Frankfurt am Main, Germany; ^7^ IQVIA, Madrid, Spain

**Keywords:** acute myeloid leukemia, hypomethylating agents, oral treatment, patient preference, treatment preference, qualitative interview

## Abstract

**Introduction:**

About half of patients with Acute Myeloid Leukemia (AML) are not eligible for Standard Induction Chemotherapy (SIC). Hypomethylating Agents (HMAs) intravenously (IV) or subcutaneously (SC) in a clinical setting are typically offered as an alternative. However, injectable HMAs may be burdensome for patients given the frequent hospital visits and side effects. This study explored patient treatment preferences for different modes of administration (MOA) and the relative importance of treatment-related characteristics that influence treatment decisions.

**Methods:**

Semi-structured 1:1 interviews were conducted with 21 adult patients with AML in Germany, the United Kingdom, and Spain, who are not eligible for SIC, had experience with HMAs or were scheduled to be treated with HMAs. After discussing their experience of living with AML and its treatments, patients were presented with hypothetical treatment scenarios to explore their preferences, and a ranking exercise to assess the relative importance of treatment characteristics that influence their treatment-decisions for AML.

**Results:**

Most patients reported an overall preference for oral administration over parenteral routes (71%), mostly due to convenience. Those preferring IV or SC routes (24%) reasoned with faster speed of action and onsite monitoring. When presented with a hypothetical situation of a patient having to choose between two AML treatments that were identical except for their MOA, the majority preferred the oral route (76%). Regarding treatment characteristics that influence treatment decisions, patients most frequently reported efficacy (86%) and side effects (62%) as important, followed by mode of administration (29%), daily life impacts (24%) and location of treatment (hospital versus home) (14%). However, only efficacy and side effects were rated as number one deciding factors (67% and 19%, respectively). Patients most frequently rated dosing regimen (33%) as least important.

**Conclusion:**

The insights gained from this study may help support patients with AML who are receiving HMA treatment instead of SIC. A potential oral HMA with similar efficacy and tolerability profiles to injectable HMAs could influence treatment decisions. Furthermore, an oral HMA treatment might decrease the burden of parenteral therapies and improve patients’ overall quality of life. However, the extent of influence MOA has on treatment decisions requires further investigation.

## 1 Introduction

Acute myeloid leukemia (AML) is a malignant disease of hemopoietic stem cells or its progenitors, which is characterized as the arrest of differentiation and aberrant proliferation of myeloid lineages (1). AML is the most common form of acute leukemia in adults, with a median age at diagnosis of 68 years (2), and the incidence is known to increase with age (3). Patients with AML are commonly treated with Standard Induction Chemotherapy (SIC) or intensive chemotherapy, which aims to eliminate the cancerous cells in the patients’ blood and bone marrow (4). However, most patients receiving SIC experience significant side effects such as fatigue, neuropathy, mouth sores, gastrointestinal problems, increased risk of infection and cognitive issues, which can be highly burdensome (5). In addition, treatment-related toxicity of SIC could lead to serious complications and early death (4, 6), therefore, more than half of patients with AML are not suitable for intensive chemotherapy regimens (7). While higher age might increase the possibility of being ineligible for SIC, age itself cannot determine treatment decisions. General health, performance status, clinically significant comorbidities and AML-related genetic abnormalities are factors considered to be associated with higher risks than benefits of SIC (8). For patients who are not eligible for SIC, less intensive, alternative treatments, such as hypomethylating agents, (HMAs) are an option (7–10). HMAs are usually administered parenterally for 5–7 days per 28-day treatment cycle, with several treatment cycles necessary to achieve a response (9, 11–13). While the side effects of HMAs are milder than those of SIC, patients might experience myelosuppression (fatigue, increased risk of infection, neutropenic fever), gastrointestinal problems (nausea, diarrhea, constipation) and - for those who receive HMAs subcutaneously - injection site-related side effects (14). Both intravenous (IV) and subcutaneous (SC) versions of HMAs have been approved for use in Europe, and current HMAs are required to be administered by healthcare professionals (12). Studies conclude that while both administration methods are effective, with no notable difference in tolerability, patients usually prefer the SC route (15, 16). The reasons are likely that the SC administration requires a shorter time commitment from the patient (17–19) and perceived to cause less pain and discomfort than the IV route (20). However, in a qualitative interview study in the context of a clinical trial, the small proportion of patients who preferred IV administration reasoned with fewer injection site reactions of IV than SC (20).

Nevertheless, both IV and SC administration HMAs necessitate frequent visits to the clinic, which requires significant time commitments from the patient and their caregivers and may be associated with both loss of productivity and other significant difficulties in carrying out daily activities (21–24). The psychological impacts of frequently visiting hospital clinics are also notable, particularly since the COVID-19 pandemic, as vulnerable patients (i.e., those who are older, frailer and/or immunocompromised) are very likely to be worried about a possible COVID-19 infection in healthcare facilities (25). Discontinuing AML treatment is likely to lead to disease progression (26). However, the burden of HMA treatment on patients and caregivers, such as the requirement of frequent hospital visits and treatment toxicity can lead to interruption of therapy – even in patients that respond to treatment (27) An orally bioavailable HMA as first line treatment for people who are not eligible to receive SIC could potentially decrease the burden of IV or SC injections and hospital visits (11, 28). To date, perspectives, and preferences of patients with AML for the different mode of administration of HMAs have not been assessed. This study aimed to explore and describe patient experiences and preferences of modes of administration (MOA) of HMAs as well as the drivers of treatment preferences among patients with AML in Germany, the United Kingdom (UK) and Spain, using one-to-one qualitative interviews.

## 2 Materials and methods

### 2.1 Design

One-to-one, semi-structured telephone interviews were conducted with patients with AML who are not eligible for SIC or was using/has used HMA for AML treatment across three countries: Germany, the UK and Spain. After discussing patients’ views and preferences on treatment settings and treatment MOA, patients were presented with a hypothetical oral treatment, known as “Product X” to review. Patients were then presented with a description of a hypothetical treatment scenario. This included a patient with AML who is facing different treatment choices. This exercise was used to gain their objective views on treatment options with different characteristics (*i.e.*, oral versus injectable HMA treatment). The hypothetical treatment scenarios presented to patients were developed by a prior, targeted literature review then reviewed by six clinicians, experienced in treating patients with AML who are not eligible for SIC or was using/has used HMA for AML treatment, *via* one-to-one qualitative interviews. Necessary updates suggested by clinicians were applied to the treatment scenarios before the patient interviews. The literature review and clinician interviews also ensured an understanding of current research and clinical management of AML and informed the development of the patient interview guides. To assess the relative importance of treatment characteristics during the patient interviews, patients were asked to talk about treatment characteristics they consider important when choosing a treatment for AML, then provide their top 3 and number 1 most important characteristics that determines their treatment decision. Patients were also asked to name characteristics previously discussed that would be less important in their decision. The study design is currently in-line with the PREFER recommendations released by the European Medicines Agency (EMA) and IMI-PREFER (29).

### 2.2 Participants

Patients were recruited from Germany, UK and Spain between January 2022 and April 2022, using a non-randomized, convenience sampling approach (30). Patients were invited to participate in the study if they fulfilled the following inclusion criteria:

- Over 18 years old
- Diagnosed with *de novo* or secondary AML
- Treatment naïve or in first line treatment
- Not eligible for SIC or was using/has used HMA for AML treatment within the last 6 months
- Receiving care for their AML in the UK, Germany, or Spain (and able to read and write in English, German, or Spanish)
- Had access to a telephone or cell phone and internet to access the screen sharing platform
- Willing and able to complete an hour-long interview and having it audio recorded.

Patients were excluded from participation if they

- Had relapsed or refractory AML
- Used oral HMAs for any other condition
- Had any condition that involved cognitive deficits or severe visual impairment that could interfere with the ability of the patient to understand or interpret the questions in the interview
- Patients that were concurrently participating in an AML clinical trial

Consented patients were asked to provide a confirmation of their diagnosis for AML and treatment *via* their physicians.

### 2.3 Data collection

Web-assisted telephone interviews, lasting 60-minutes on average, were conducted by moderators who spoke the local languages (German, English or Spanish) and guided by a brief, semi-structured discussion guide, and a short set of slides of hypothetical scenarios. The discussion guide included questions on 1) patient burden of AML and its treatment including symptoms and daily life impacts, 2) patients’ overall treatment experiences and preferences regarding location and MOA of treatment; 3) patients’ perspectives on a hypothetical oral treatment that were presented as “Product X”; 4) patients’ objective views on treatment scenarios a hypothetical patient had to choose from; and 5) drivers of treatment preference. The moderator used the interview guide to tailor the discussion but also asked follow-up questions as appropriate. (Please see **Supplementary Material 1** for the interview guide). The interviews were audio-recorded with interviewee permissions and transcripts were created verbatim from the recordings. Spanish and German interviews were transcribed to local language before being translated to English. Transcripts were analyzed using MAXQDA, a qualitative analysis software (31), with a pre-developed coding framework, which was used to identify key themes. During analysis, this framework was expanded with spontaneously mentioned as well as probed themes emerging from the data.

## 3 Results

### 3.1 Sample characteristics

The study planned to recruit 45 patients with AML in Germany, UK, and Spain (n=15 per country). However, difficulties with recruitment were experienced in the UK and Spain, likely because patients with AML who are not eligible for SIC or was using/has used HMA for AML treatment are typically older, with lower performance status and clinically significant comorbidities. Prospective participants might have felt too unwell or uncomfortable to partake in the interviews. While recruitment target was reached in Germany (n=15), the final numbers were lower than expected in the UK (n=4) and Spain (n=2). However, as the study objective was to assess overall patient preferences, this patient sample size was deemed adequate to fulfil this objective (32).

Patients’ sociodemographic and clinical characteristics are presented in **Table 1** and **Table 2**. Twenty-one patients with AML (n=15 from Germany, n=4 from the UK and n=2 from Spain) were interviewed for this study between the ages of 33 and 72 years, with an average age of 52 years (median: 51 years). Patients were mostly female (n=14; 67%) and white (n=19; 90%) with one patient each reported being Asian (5%) and mixed ethnicity (5%). While the typical patient with AML is expected to be retired due to AML’s higher prevalence in the older population (2) only 14% of the current study sample reported being retired (n=3) due to the lower than usual average age. Nearly half of the patients did not work due to disability (n=10; 47%), four patients had a part time job (19%), three patients were unemployed (14%), and one patient worked full time (5%).

**Table 1:** Sociodemographic characteristics of patients (N=21).

	Total (N=21) n (%)	Germany (n=15) n (%)	UK* (n=4) n (%)	Spain* (n=2) n (%)
Age (Years)				
18-34	1 (5)	0 (0)	1 (25)	0 (0)
35-44	6 (29)	5 (33)	1 (25)	0 (0)
45-54	7 (33)	4 (27)	1 (25)	2 (100)
55-64	5 (24)	4 (27)	1 (25)	0 (0)
65+	2 (10)	2 (13)	0 (0)	0 (0)
Gender				
Male	7 (33)	5 (33)	1 (25)	1 (50)
Female	14 (67)	10 (67)	3 (75)	1 (50)
Ethnicity				
White	19 (90)	14 (93)	3 (75)	2 (100)
Asian	1 (5)	0 (0)	1 (25)	0 (0)
Mixed/Multiple Ethnic Groups	1 (5)	1 (7)	0 (0)	0 (0)
Employment Status				
Employed Full-time	1 (5)	0 (0)	1 (25)	0 (0)
Employed Part-time	4 (19)	4 (27)	0 (0)	0 (0)
Unemployed	3 (14)	2 (13)	1 (25)	0 (0)
Retired	3 (14)	2 (13)	1 (25)	0 (0)
On disability	10 (48)	7 (47)	1 (25)	2 (100)

* Recruitment difficulties in the UK and Spain resulted in lower-than-expected sample size in these countries as described in limitations.

**Table 2:** Clinical characteristics of patients (N=21).

	Total (N=21) n (%)	Germany (n=15) n (%)	UK* (n=4) n (%)	Spain* (n=2) n (%)
Activity Rating^1^				
Normal with no limitations	1 (5)	0 (0)	1 (25)	0 (0)
Not my normal self, but able to be up and about with fairly normal activities	19 (90)	15 (100)	2 (50)	2 (100)
Not feeling up to most things, but in bed or chair less than half of the day	1 (5)	0 (0)	1 (25)	0 (0)
Able to do little activity and spend most of the day in bed or chair	0 (0)	0 (0)	0 (0)	0 (0)
Pretty much bedridden, rarely out of bed	0 (0)	0 (0)	0 (0)	0 (0)
Time Since Diagnosis				
≤ 6 months	14 (67)	14 (93)	0 (0)	0 (0)
6 months – 1 year	1 (5)	1 (7)	0 (0)	0 (0)
≥ 1 year	6 (29)	0 (0)	4 (100)	2 (100)
Treatment status				
Currently receiving treatment	11 (52)	11 (73)	0 (0)	0 (0)
Not currently receiving treatment (but received treatment in the past)	6 (29)	0 (0)	4 (100)	2 (100)
Treatment naïve	4 (19)	4 (27)	0 (0)	0 (0)
Treatment MOA^2^				
Oral only	0 (0)	0 (0)	0 (0)	0 (0)
SC only	1 (5)	0 (0)	1 (25)	0 (0)
IV only	2 (10)	0 (0)	1 (25)	1 (50)
Combination	12 (57)	11 (73)	1 (25)	0 (0)
* Oral* ^3^ *+ SC*	8 (38)	8 (53)	0 (0)	0 (0)
* Oral + IV*	3 (14)	2 (13)	1 (25)	0 (0)
* SC+ IV*	0 (0)	0 (0)	0 (0)	0 (0)
* Oral + IV+ SC*	1 (5)	1 (7)	0 (0)	0 (0)
Missing^4^	6 (29)	4 (27)	1 (25)	1 (50)

MOA, Mode of administration; SC, Subcutaneous; IV, Intravenous.

* Recruitment difficulties in the UK and Spain resulted in lower-than-expected sample size in these countries as described in limitations.

^1^ Self-reported using the ECOG rating scale ranging from 0-4, with a score of 0: Normal with no limitations, and a score of 4: Pretty much bed ridden, rarely out of bed.

^2^ Includes current treatment and prior treatment of those who were treated in the past but did not receive treatment at the time of screening.

^3^ Oral treatments received in combination were not HMA.

^4^ Includes treatment naïve patients and patients that did not provide. MOA data of their treatment.

At screening, patients were asked to rate their activity levels, using The Eastern Cooperative Oncology Group (ECOG) rating scale (33) The majority of patients (n=19; 90%) rated their activity level as a score of 1: *“Not my normal self, but able to be up and about with fairly normal activities”*. About two-third of the patients (n=14; 67%) received their AML diagnosis less than 6 months prior to screening. Only one patient (5%) was diagnosed 6-12 months prior to the interview and six patients (29%) were diagnosed more than 1 year prior to interview. Eleven patients (52%) reported receiving treatment for their AML at the time of screening. Out of the remaining ten patients (48%) who reported not currently being on treatment, six stated previously receiving treatment for AML and four patients were treatment naïve. Regarding MOA of treatments patients received currently or previously, most patients (n=12; 57%) reported combination treatments (parenteral HMA and oral non-HMA), one patient received subcutaneous treatment only (5%), and two received intravenous treatment only (10%). No data was available on MOA of current or past treatment for six patients including four treatment naïve patients and two patients who did not answer this question on the screening form.

### 3.2 Patient burden of AML

Patients were asked to describe their day-to-day experiences of living with AML since they have been diagnosed, including the burden of receiving treatment for AML. Fatigue was the most frequently reported symptom (n=11; 52%). For instance, one patient noted, *“There are days when I don’t feel like getting out of bed, I simply feel tired. Or I’m up for 3 hours and I have to lie down again.” (P20, Germany).* Other symptoms mentioned by patients included gastrointestinal issues (diarrhea, acid reflux, n=3; 14%), eczema/skin irritation (n=2; 10%), pain (n=2; 10%), nausea (n=2; 10%), brain fog (n=1; 5%), dizziness (n=1; 5%) and headaches (n=1; 5%). According to the patients, the areas of daily life that were mostly negatively impacted by AML and its treatment were work/employment (n=12; 57%), usual daily activities (*i.e.*, cooking, cleaning, shopping, driving) (n=9; 43%) and emotional functioning (*i.e.*, anxiety, worries) (n=6; 29%) were most frequently affected by AML or its treatment. Several patients commented that they were unable to return to the workplace due to the associated illness and related symptoms (e.g., fatigue) and risk of being exposed to infection due to being immunocompromised. For example, one patient noted, *“I can’t work at the moment because I often feel nauseous. In that case it gets so bad that I have to sit down.” (P5, Germany).* About a quarter of the patients also described impacts on their social functioning (n=5; 24%), such as not being able to attend social activities, seeing friends or going on trips and their outlook on life (n=5; 24%). For instance, one patient noted, *“A whole new perspective on life. Most of it now is all positive, which is good. To start off, definitely it was a struggle.” (P8, UK)*. Other impacts patients mentioned were physical functioning, for example climbing stairs (n=4; 19%), finances, mostly due to loss of employment (n=3;14%) and impacts on their physical appearance (n=2; 10%) and sexual functioning (n=1; 5%). The full list of symptoms and daily life impacts in total and per country are presented in **Table 3**.

**Table 3:** Symptoms and daily life impacts of patients with AML (N=21).

	Total (N=21) n (%)	Germany (n=15) n (%)	UK* (n=4) n (%)	Spain* (n=2) n (%)
Symptoms				
Fatigue	11 (52)	7 (47)	2 (50)	2 (100)
Gastrointestinal (Diarrhea/acid reflux)	3 (14)	2 (13)	0 (0)	1 (50)
Eczema/skin irritation	2 (10)	1 (7)	0 (0)	1 (50)
Pain	2 (10)	0 (0)	0 (0)	2 (100)
Nausea	2 (10)	2 (13)	0 (0)	0 (0)
Cognitive (Brain fog)	1 (5)	0 (0)	0 (0)	1 (50)
Dizzy spells	1 (5)	1 (7)	0 (0)	0 (0)
Headaches	1 (5)	1 (7)	0 (0)	0 (0)
Daily life impacts				
Work/Employment	12 (57)	7 (47)	3 (75)	2 (100)
Daily Activities	9 (43)	7 (47)	1 (25)	1 (50)
Emotional function	6 (29)	4 (27)	2 (50)	0 (0)
Outlook on life/Plans for the future	5 (24)	3 (20)	2 (50)	0 (0)
Social Function	5 (24)	3 (20)	1 (25)	1 (50)
Physical Function	4 (19)	2 (13)	2 (50)	0 (0)
Financial Impact	3 (14)	0 (0)	2 (50)	1 (50)
Physical appearance	2 (10)	0 (0)	0 (0)	2 (100)
Sexual Function	1 (5)	0 (0)	0 (0)	1 (50)

* Recruitment difficulties in the UK and Spain resulted in lower-than-expected sample size in these countries as described in limitations.

### 3.3 Overall treatment experience and preferences

#### 3.3.1 Benefits and disadvantages

Patients also discussed their overall experiences of receiving treatment for AML and the potential benefits of receiving treatments in different locations (home versus clinic) and *via* different MOA. While all best efforts were made to elicit patient views on benefits and disadvantages of treatment setting and MOA from the full sample, only 11 patients provided answers. Six patients were not able to identify benefits and disadvantages and four patients were not asked due to time constraints. However, the reported percentages are calculated based on the full sample (n=21).

The benefits of being treated at home was described as comfortable (n=3; 14%) and convenient (n=1; 5%). For example, one patient noted, *“I’d rather be at home than sitting at the doctors for an hour or 3 hours for an infusion.” (P21, Germany).* However, a patient also stated that less monitoring by medical professionals (n=1; 5%) could be disadvantageous and noted previous occasions of rehospitalization following an early discharge. Talking about benefits of a clinic setting, patients mentioned more monitoring (n=4; 19%). For instance, one patient noted, *“By having the treatment at the hospital and then being monitored, that also gives you a sense of security.” (P20, Germany)* while inconvenience (n=5; 24%), anxiety about a possible COVID infection (n=4; 19%) and the general clinic environment (n=2; 10%) (*i.e.*, difficult to rest, isolation) were noted as disadvantages.

Regarding benefits of the oral administration, the ability to administer the treatment at home (n=2, 10%), convenience (n=1, 5%) and minimal side effects (n=1, 5%) were advantages. For example, one patient mentioned, *“I can take it* [oral medication] *with me if I go somewhere, I can always take it with the liquid I am drinking. [ … ] it is simple, fast and easy to take.” (P12, Germany)*. However, the inconvenience of structuring their life around taking pills (n=2, 10%) and taste of medication (n=1; 5%) were reported as disadvantages for oral treatment. Patients mentioned higher perceived efficacy as benefits of IV. For instance, a patient stated, *“I get the impression that by putting the medication into the vein, it does more damage to the disease.” (P19, Spain).* However, side effects (n=1; 5%) and discomfort (n=1; 5%) of the IV administration were disadvantages. Finally, for SC administration, patients found that the simplicity (n=1; 5%) and lower frequency of administration (n=1; 5%) were advantages. For example, *“It* [SC injection] *doesn’t have to go through the mouth and stomach because sometimes that can be a little bit inflamed and it’s simple, the injections are done. So it’s easier for me.” (P11, Germany)*. Side effects (*i.e.*, bruising) (n=1; 5%) and difficulty of administration (n=2; 10%) were disadvantages reported for SC treatment.

#### 3.3.2 Overall preferences of MOA

All patients were asked about their preferences for different MOA overall and most patients (n=15; 71%) preferred oral over the IV and SC routes. Three patients (14%) favored the IV administration, two patients (10%) the SC administration over the others and one patient was unable to decide on a preferred MOA. Preferences of MOA by country are shown in **Table 4**.

**Table 4:** Patient preferences of MOA of treatment for AML.

	Total (N=21) n (%)	Germany (n=15) n (%)	UK* (n=4) n (%)	Spain* (n=2) n (%)
Oral	15 (71)	12 (80)	2 (50)	1 (50)
IV	3 (14)	0 (0)	2 (50)	1 (50)
SC	2 (10)	2 (13)	0 (0)	0 (0)
Missing	1 (5)	1 (7)	0 (0)	0 (0)

* Recruitment difficulties in the UK and Spain resulted in lower-than-expected sample size in these countries as described in limitations.

### 3.4 Perspectives on a hypothetical, oral treatment

Patients were presented with a description of a hypothetical, oral HMA treatment, “Product X”, which was verbally described to them by the moderator as follow:

*Imagine you are offered an oral treatment for your AML. It is a pill. You would need to take this treatment for 5 consecutive days, then you would have 23 days without treatment, then you would take it again for 5 days, followed by 23 days with no treatment, and so on. So, it would be taken for 5 consecutive days in 28-day cycles. You would need to take the pill at the same time each day and you could not eat for 2 hours before taking it, or for 2 hours after taking it. You could take this medication at home*.

Patients were asked to share whether or not this treatment would appeal to them and what kind of challenges they think they might face when taking it as prescribed. Overall, all patients (n=21; 100%) found the profile of Product X appealing. The majority (n=16; 76%) stated that the treatment regimen was the key contributor for the appeal, including the few days of administration followed by a longer break, the possibility to take it at home and that no other treatment would be needed. For example, one patient reported,*”That would be the only medication, which doesn’t sound so bad at all. One step that is totally gone.” (P4, Germany)*. About a third of the patients (n=8; 38%) listed convenience as a reason that made Product X appealing, two patients (10%) reasoned with the fact that it is an oral product and one patient (5%) mentioned that Product X as a new treatment was favorable.

Most patients (n=16; 76%) reported that they would have no problems taking Product X the way it was presented to be prescribed. For instance, one patient noted, *“I don’t think that’s particularly onerous. It’s only one tablet and you’re talking about 4 hours where you’re not eating within a 24-hour period, so that sounds quite easy to fit into your daily schedule.” (P7, UK).* However, a few patients stated that the strict schedule of taking the hypothetical drug for 5 consecutive days at the same time each day (n=3; 14%) and not eating for 2 hours before and after (n=2; 10%) could be potential challenges. For example, a patient mentioned, *“If I take them daily, maybe less so, but if I then take them for 5 days, then stop for what would be maybe 24 or 25 days [ … ] that would have to be quite accurate. That would perhaps be the only problem” (P4, Germany).* Furthermore, two patients pointed out potential challenges that were not described in the presentation of Product X. One patient (5%) was not sure whether Product X could be taken with water as the description did not allow eating before and after and the other patient assumed that this treatment would have side effect which raised concerns about its impact on employment (5%). This patient stated, “*I don’t want to be unemployed until the end of my life. That doesn’t work with this treatment. And you feel pretty awful, to put it mildly” (P3, Germany).*

Following the discussion on the oral Product X, patients were asked to think about the same treatment but now receiving it *via* parenteral routes, then decide which MOA of Product X they would prefer. The moderator verbally provided the following scenario:

*I would like you to think about this oral treatment and compare it to a SC and an IV version of the same treatment. In all cases it would be taken for 5 to 7 days with a break of 23 days before starting again; for the IV and SC this would be done in the hospital. It would take about 1-3 hours, including the injection time and the monitoring time after the injection. For the oral pill you can take it at home, and the physician may decide to reduce hospital/treatment center visits. In the case of the pill you would not be able to eat for 2 hours before and 2 hours after. There is no such restriction on the IV/SC*.

Nearly all patients (n=20; 95%) stated that they preferred the oral Product X, compared to either an IV or SC version of the same treatment. Their preferences were mostly due to not wanting to spend time for traveling and waiting at the hospital, and difficulties with organizing transportation. For example, a patient pointed out, *“You have to factor in the trip to the hospital [ … ]. Of course, logistically that’s all a bit more stressful than if I just take a tablet from home.” (P17, Germany).* However, one patient (5%) preferred the IV or SC version of the same treatment, noting the advantage of being monitored by medical professionals. This patient stated, *“Let’s put it this way: something happens to me, I’m alone, my husband is at* [work] *[ … ]. If I take the tablet here at home for 7 days and something happens to me, I’m alone and there is no one to take care of me.” (P14, Germany).*

### 3.5 Comparing treatment scenarios

**Figure 1:**
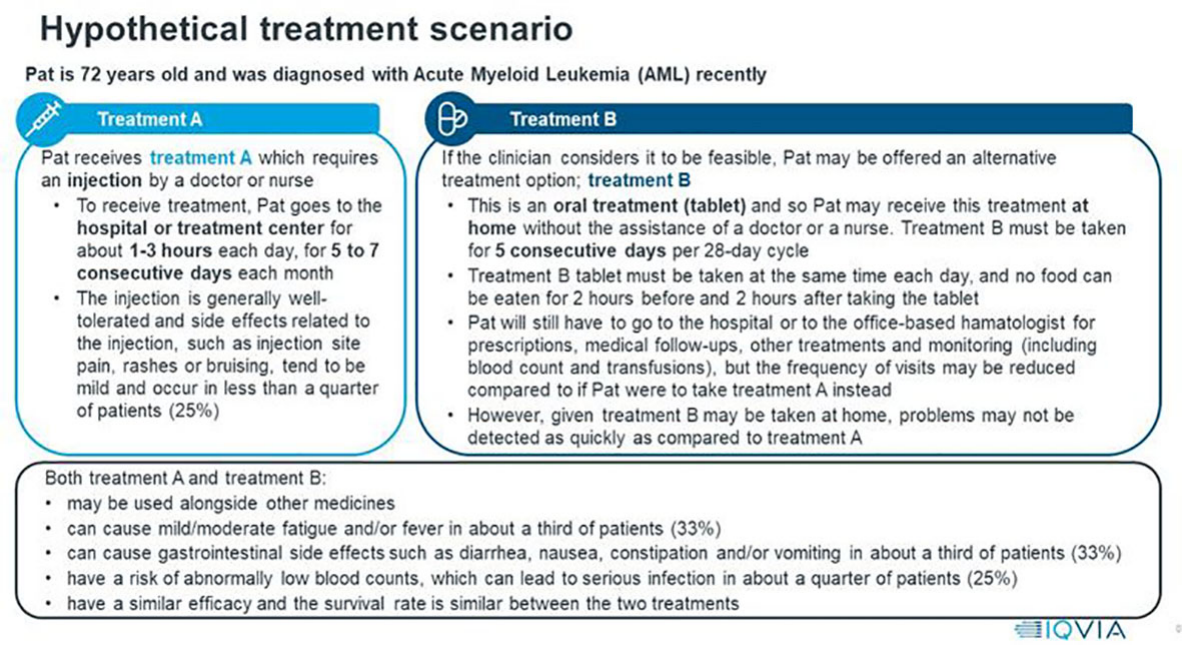
Hypothetical AML treatment scenarios presented to patients.

Following the discussion on Product X, patients reviewed hypothetical treatment scenarios presented to them as a choice of a fictional character, named “Pat”, who is 72 years old and has AML. “Pat” is facing two treatment options: a currently available treatment, with an injectable HMA (Treatment A), and an alternative treatment, which is an oral HMA (Treatment B, identical to Product X presented earlier in the interview). Patients were asked to review the scenarios, then comment on the clarity and understanding of Treatment A and Treatment B, as well as their preference between the two treatments. The scenarios presented to patients are shown in **Figure 1**.

All patients (n=21; 100%) stated that they found the scenarios to be clear, with the information presented clearly and the vast majority (n=20; 95%) reported understanding the descriptions and the distinctions between Treatment A and Treatment B. Most patients preferred Treatment B (oral HMA) (n=16; 76%), whereas three patients (14%) chose Treatment A (injectable HMA). Two patients (10%) were unable to make a treatment decision, despite being prompted from the moderator. Patients who chose Treatment B listed a variety of reasons including convenience of being able to administer the treatment at home (n=10; 48%), dislike of clinic environment (*i.e.*, travel time, waiting time) (n=2; 10%), oral administration (n=2; 10%) and reduced risk of infection that might be caught in the clinic (n=1; 5%). Patients choosing Treatment A noted that they would prefer to be at the hospital and receive more monitoring (n=2; 10%) and support in case there were any complications (n=1; 5%). For instance, a patient mentioned, *“The good thing about it is being onsite should anything go wrong in terms of side effects.” (P2, UK).* One patient (5%) switched their preference while discussing the two options. Initially, the patient chose Treatment A; however, when weighing up the different characteristics of the treatments and discussing what matters most to them, the patient decided to change their preference. Explaining the final decision, the patient stated that receiving the treatment at home would save time and reduce burden on having to rely on others to travel to the hospital. This patient stated, *“Looking at Treatment B [ … ] I guess, you could say for those 5 days, I could’ve stayed at home [ … ]. Whereas I am tied to going for the 5 consecutive days for the injection plus any other visits that I may have had to do.” (P6, UK)*

When asked to reflect on the most important features of the two scenarios, patients mostly considered similar characteristics for Treatment A and B, however, the importance of these in their treatment decision overall seemed to be different. For Treatment A, monitoring was considered important more frequently than for Treatment B (n=7; 33% and n=3; 14% respectively) however, location of treatment (hospital versus home) was a more frequently mentioned important feature for Treatment B compared to Treatment A (n=16; 76% and n=4; 19% respectively). Length of administration (n=4; 19%) and side effects (n=3; 20%) were mentioned by the same number of patients as important characteristics for both treatments. In addition, MOA was mentioned by three patients (14%) as an important feature of Treatment B to influence their decision, while only one patient thought about this for Treatment A. **Table 5** lists all treatment characteristics patients reported considering during treatment decision-making in the hypothetical situation.

**Table 5:** Treatment characteristics patients considered for Treatment A and B.

	Treatment A (Injectable HMA)				Treatment B (Oral HMA)			
	Total (N=21) n (%)	Germany (n=15) n (%)	UK* (n=4) n (%)	Spain* (n=2) n (%)	Total (N=21) n (%)	Germany (n=15) n (%)	UK* (n=4) n (%)	Spain* (n=2) n (%)
Location of administration	4 (19)	3 (20)	1 (25)	0 (0)	16 (76)	10 (67)	4 (100)	2 (100)
Length of administration	4 (19)	3 (20)	1 (25)	0 (0)	4 (19)	3 (20)	1 (25)	0 (0)
Monitoring	7 (33)	4 (27)	1 (25)	2 (100)	3 (14)	3 (20)	0 (0)	0 (0)
Side effects	3 (14)	2 (13)	1 (25)	0 (0)	3 (14)	3 (20)	0 (0)	0 (0)
Method of administration	1 (5)	0 (0)	1 (25)	0 (0)	3 (14)	1 (7)	1 (25)	1 (50)
Time of day for administration	0 (0)	0 (0)	0 (0)	0 (0)	1 (5)	1 (7)	0 (0)	0 (0)
Frequency of administration	–	–	–	–	1 (5)	0 (0)	0 (0)	1 (50)
Travel	1 (5)	0 (0)	0 (0)	0 (0)	–	–	–	–
Not mentioned	6 (29)	4 (27)	2 (50)	0 (0)	–	–	–	–

* Recruitment difficulties in the UK and Spain resulted in lower-than-expected sample size in these countries as described in limitations.

Patients were also asked if they saw any specific side effects in the description of the treatments which would have influenced their treatment decision. Patients most frequently (n=8; 38%) noted that there were no specific side effects listed that they could not tolerate. For example, one patient stated, *“I think the side effects are fully within expectations. It’s nothing that would scare me now.” (P15, Germany).* Those talking about specific side effects they wished to avoid most frequently mentioned fever (n=4; 19%), serious infections (n=4; 19%), diarrhea (n=3; 14%) and constipation (n=2; 10%). Two patients (10%) reported not wanting to have any side effects.

### 3.6 Drivers of treatment preference

**Table 6** displays the list of most important treatment characteristics patients reported considering, and shows the relative importance of these when deciding about treatments.

**Table 6:** Important treatment characteristics in decision-making for AML treatment.

	Total (N=21) n (%)	Germany (n=15) n (%)1	UK* (n=4) n (%)	Spain* (n=2) n (%)
Efficacy	18 (86)	13 (87)	3 (75)	2 (100)
Side effects	13 (62)	9 (60)	2 (50)	2 (100)
MOA	6 (29)	5 (33)	0 (0)	1 (50)
Daily life impact	5 (24)	4 (27)	0 (0)	0 (50)
Therapy duration	5 (24)	5 (33)	0 (0)	0 (0)
Time spent in hospital	3 (14)	0 (0)	1 (25)	2 (100)
Location of treatment (hospital vs home)	3 (14)	1 (7)	0 (0)	2 (100)
Financial impact	3 (14)	2 (13)	0 (0)	1 (50)
Dosing regimen (frequency of administration)	2 (10)	1 (7)	0 (0)	1 (50)
Contraindications	2 (10)	2 (13)	0 (0)	0 (0)

MOA, Mode of administration.

* Recruitment difficulties in the UK and Spain resulted in lower-than-expected sample size in these countries as described in limitations.

Patients occasionally ranked characteristics that were not previously mentioned as important.

Patients commonly listed more than one characteristic; therefore, percentages may not add up to 100.

When asked to discuss treatment characteristics that were important for treatment decisions, efficacy and side effects were mentioned by the majority patients (n=18; 86% and n=13; 62% respectively). About a quarter of the patients mentioned MOA (n=6; 29%), daily life impacts (n=5; 24%) and therapy duration (n=5; 24%) as important characteristics. Other important treatment characteristics reported were time spent in hospital (n=3; 14%), location of treatment (hospital versus home, n=3; 14%), financial impacts of treatment (n=3; 14%), dosing regimen (frequency of administration) (n=2; 10%) and contraindications (n=2; 10%).

In the ranking exercise (**Table 7**), efficacy was ranked in the top 3 most important characteristics by fifteen patients (71%) and over two-third of the sample considered this as their number one characteristic (n=14; 67%). For instance, one patient mentioned, *“What are the chances of survival. I think that’s the first thing. It’s really crucial if he* [clinician] *tells me that I’m going to pump you full of medication and extend your life by 6 months. The decision would then be different if he said we have good chances of survival, we really fight for it now.” (P9, Germany).* The majority of patients ranked side effects in their top 3 (n=13; 62%) and four (19%) stated that this would be the number one most important characteristic in deciding for or against a treatment. For example, one patient noted, *“The only thing that would make me choose differently between the two is serious side effects and less serious side effects.”* (P2, UK). While only efficacy and side effects were rated as number one most important treatment characteristic by patients, several other treatment characteristics were listed as top 3 by importance. Therapy duration was listed in top 3 by six patients (29%), mode of administration by five (24%), daily life impacts by three patients (14%) and dosing regimen by two patients (10%). For instance, one patient reported, *“The general limitation of everyday life [ … ] of course that is very important to me, but it also doesn’t help me if I can then enjoy my everyday life, but the medication either has to be taken forever, or does not work at all.” (P13, Germany).* Furthermore, frequency of hospitalization, location of treatment (hospital versus home), progression-free survival and other patients’ experiences were listed in top 3 buy one patient (5%) each.

**Table 7:** Relative importance of treatment characteristics in decision-making.

	Total (N=21) n (%)	Germany (n=15) n (%)1	UK* (n=4) n (%)	Spain* (n=2) n (%)
Efficacy				
Ranked in top 3	15 (71)	10 (48)	3 (75)	2 (100)
Ranked 1^st^	14 (67)	9 (43)	3 (75)	2 (100)
Side effects				
Ranked in top 3	13 (62)	9 (43)	4 (100)	0 (0)
Ranked 1^st^	4 (19)	3 (14)	1 (25)	0 (0)
Therapy duration				
Ranked in top 3	6 (29)	5 (24)	1 (25)	0 (0)
Ranked 1^st^	0 (0)	0 (0)	0 (0)	0 (0)
MOA				
Ranked in top 3	5 (24)	4 (19)	1 (25)	0 (0)
Ranked 1^st^	0 (0)	0 (0)	0 (0)	0 (0)
Financial impact				
Ranked in top 3	3 (14)	3 (14)	0 (0)	0 (0)
Ranked 1^st^	0 (0)	0 (0)	0 (0)	0 (0)
Daily life impact				
Ranked in top 3	3 (14)	1 (5)	1 (25)	1 (50)
Ranked 1^st^	0 (0)	0 (0)	0 (0)	0 (0)
Dosing regimen (frequency of administration)				
Ranked in top 3	2 (10)	2 (13)	0 (0)	0 (0)
Ranked 1^st^	0 (0)	0 (0)	0 (0)	0 (0)
Frequency of hospitalization				
Ranked in top 3	1 (5)	0 (0)	1 (25)	0 (0)
Ranked 1^st^	0 (0)	0 (0)	0 (0)	0 (0)
Location of treatment (hospital vs home)				
Ranked in top 3	1 (5)	0 (0)	1 (25)	0 (0)
Ranked 1^st^	0 (0)	0 (0)	0 (0)	0 (0)
Progression free survival				
Ranked in top 3	1 (5)	1 (7)	0 (0)	0 (0)
Ranked 1^st^	0 (0)	1 (7)	0 (0)	0 (0)
Other patients’ experiences with treatment				
Ranked in top 3	0 (0)	0 (0)	0 (0)	0 (0)
Ranked 1^st^	0 (0)	0 (0)	0 (0)	0 (0)

MOA, Mode of administration.

* Recruitment difficulties in the UK and Spain resulted in lower-than-expected sample size in these countries as described in limitations.

Patients occasionally ranked characteristics that were not previously mentioned as important.

Patients commonly listed more than one characteristic; therefore, percentages may not add up to 100.

When asked about characteristics they considered to be less important in treatment decision-making, none of the patients mentioned efficacy or side effects. The treatment characteristic patients most frequently thought less important in treatment decisions was dosing regimen (n=7; 33%) followed by mode of administration (n=3; 14%), time spent in hospital (n=3; 14%) and finances (n=3; 14%). Daily life impacts, physical location of treatment and therapy duration were mentioned by two participants each (10%) as less important characteristics.

## 4 Discussion

The overall objective of this study was to explore and describe patient experiences living with AML and their preferences of MOA treatments among patients with AML who are not eligible for SIC or was using/has used HMA for AML treatments. Our findings showed patients most commonly reported fatigue, gastrointestinal symptoms, eczema/skin irritation and nausea, ‘brain fog’, headaches and dizziness (20). Regarding impacts of AML and its treatment, patients most commonly mentioned impacts on their work/employment, their ability to carry out daily activities and their emotional functioning, consistent with previous studies (34–37). Comfort and convenience were also the most commonly mentioned benefits of receiving treatment at home, consistent with other studies, while less monitoring was noted as a disadvantage (38). Monitoring treatments was a further benefit mentioned by patients, previously noted in the literature how monitoring treatments by either a nurse or doctor may help facilitate adherence (39). On the other hand, the inconvenience of traveling to the clinic, being anxious of the possible consequences of catching COVID-19 in their condition and the discomfort of clinic environment were listed as disadvantages. Patients frequently highlighted the ability to administer the treatment at home as the most notable benefit of oral treatment, followed by convenience and minimal side effects. A few patients also mentioned the inconvenience of remembering to take a tablet every day as a disadvantage (40). When discussing parenteral treatments, some patients perceived the IV MOA to be more efficient than other routes, while others pointed out the side effects and discomfort of receiving an IV injection as a disadvantage (38). Lower frequency and simplicity of administration were mentioned as benefits of SC MOA and difficulty of administration and the side effects of administering the treatment (e.g., bruising) were noted as disadvantages (20).

When discussing preferences for different MOA, most patients reported a preference for receiving an oral treatment over IV and SC administration, in line with previous findings (38, 41). When presented with a hypothetical oral HMA treatment “Product X”, all patients found the oral profile highly appealing and nearly all chose this oral version of Product X compared to the IV or SC route of the same treatment. Similarly, when being presented with a treatment scenario where a hypothetical patient had to choose from an injectable and an oral HMA profile, both with the same efficacy and side effect characteristics, most patients preferred the oral administration. This finding replicates previous literature revealing preference for oral MOA (25, 38, 41). Convenience of home administration was the most frequently mentioned benefit of a hypothetical oral HMA, as they require less preparation and are quick to administer in comparison to receiving an SC or IV treatment (17–19). The ability to take a tablet in their home may also allow patients to reduce the number of visits to the hospital, possibly to reduce exposure to infection such as COVID-19 (21, 25, 38). The few patients who chose an injectable route in the treatment scenario exercise explained their choice with better monitoring in the clinic setting, however this can potentially be done at home using telemedicine devices, where real-time data is collected for remote monitoring, which can lead to improved patient quality of life, better symptom control and adherence and decreased emergency room visits (42–46). Lastly, when describing different characteristics influencing treatment decisions, most patients listed efficacy and side effects of treatment as important treatment characteristics. Patients also most commonly noted dosing regimen as least important for treatment decision-making. This suggests, that while patients consistently preferred an oral, home administered treatment in each exercise provided, efficacy and tolerability continue to be the most important characteristics that patients consider for treatment decision-making (38).

### 4.1 Limitations and future research

Given the hypothetical nature of the preference exercises shared during the qualitative interviews, stated preferences may differ to patients’ actual preferences. However, since patients were asked to comment on their overall preferences for different MOA of AML treatments, including their experiences as well as hypothetical situations, it is likely, that both stated and actual preferences were captured in the current study. However, due to the low sample size of a qualitative study, patients’ overall stated preferences and preferred treatment characteristics may not be the same in real-world clinical practice, therefore, the results of the current study may not be generalizable.

The study could not recruit the planned number of patients with AML in each target country and the final sample included n=4 patients in the UK and n=2 patients in Spain instead of n=15 in each. However, as the study objective was to assess overall patient preferences, this sample size was deemed adequate to fulfil this objective (32). Patients being recruited from three different countries might have also caused bias due to differences in provision and treatment availability across healthcare systems. Moreover, German patients were recruited from one region in Germany compared to recruitment methods used in the UK and Spain. The healthcare system can provide capacity and can act as an important driver for treatment decision-making, as it can determine whether patients have available resources for treatment (47). Since there was an imbalance of patient numbers across the three countries with German patients dominating the sample, it is likely that the final results represent the German healthcare system more than the UK or Spanish healthcare systems. For example, the importance patients placed on financial impacts of treatment might have been impacted by different payer methods across the countries. While there were no immediate differences in preferences noted across the three countries, more investigation needed to determine, how the healthcare system of these countries might impact patients’ preferences for different MOA.

Moreover, saturation was not reached for impacts of AML as two impacts (physical appearance and sex life) were only mentioned in the final interviews. This was to be expected given the lower-than-expected sample size and the heterogeneous nature of the patient sample. Nevertheless, both impacts were mentioned in the Spanish sample, indicating that saturation might have been reached in the combined UK and German populations. In addition, themes mentioned in the earlier interviews were being replicated across the sample, indicating a level of completeness and that a sufficient sample size was included to assess overall preferences (48).

Furthermore, there were limitations observed regarding the characteristics of the sample. Typically, patients with AML who are not eligible for SIC or was using/has used HMA for AML treatment are over 65 years old, have lower performance status, or have clinically significant comorbidities (49). The median age of the current sample, however, was 51 years, ranging between 35 and 64 years, and most participants self-reported an ECOG performance status score of 1, indicating minimal or no mobility issues (*i.e.*, traveling to hospital). Therefore, the sample of this study did not align well with the typical characteristics of patients with AML who are not eligible for SIC or was using/has used HMA for AML treatments, so findings may not be fully representative and potentially limit the generalizability to older patients. While all efforts were made to recruit patients with more typical characteristics, challenges were experienced due to this patient population being frailer, struggling with fatigue and other symptoms and less able or willing to participate in interviews requiring significant efforts.

Finally, while the study aimed to capture patient preferences using explorative qualitative methods, to better estimate and quantify patient preferences, a stated preference methodology, that is suitable to generate preference weights would need to be used, such as a Discrete Choice Experiment (DCE) (50). While the current sample size, designed for a qualitative interview study, is not suitable to conduct a quantitative preference research (51), the current findings could potentially contribute to developing attributes for a future DCE (50, 52). However, given the difficulties with recruiting the patients of interest, it might be challenging to reach a suitable sample size for a DCE study.

Several implications could be drawn from this work. Patients preferred an oral MOA compared to parenteral methods and repeatedly reported the convenience of receiving treatment at home as an advantage of oral administration. While efficacy and side effects were considered most important in making a treatment decision, when efficacy and tolerability profiles are identical for treatments with different MOA, patients prefer the oral administration over parenteral routes. The insights gained from this study supports the need for an oral HMA treatment option that could improve the adherence and overall quality of life of patients with AML.

## Data availability statement

The raw de-identified data supporting the conclusions of this article will be made available by the authors upon request.

## Ethics statement

The studies involving human participants were reviewed and approved by WCG IRB 1019 39th Ave., SE Suite 120 Puyallup, WA 98374. The study protocol, recruitment materials, informed consent, and interview guide were reviewed and approved by the governing independent review board prior to implementation. The patients/participants provided their written informed consent to participate in this study.

## Author contributions

AD: Study management, intellectual input on methodology, review of study protocol and final report, interpretation of results. DB: Intellectual input on methodology and study design, review of study protocol and final report, interpretation of results. AE: Study management, review of final report and interpretation of results. LB: Study management and recruitment, conduction of analysis, interpretation of results, write-up of final report and review. IA: Support of study management and recruitment, conduction of analysis, interpretation of results, write-up of final report and review. SB: intellectual input on methodology and study design, development of study protocol and materials. SP: conduction of analysis, interpretation of results, write-up of final report and review. AMR-L: intellectual lead of study design and methodology, development of study protocol, development and oversight of study materials, review and guidance of analyses, interpretation of results, review of final report. All authors contributed to the article and approved the submitted version.
