# Supplementary material for: Exploring preferences of different modes of administration of hypomethylating agent treatments among patients with acute myeloid leukemia
Source: Front Oncol. 2023 May 8;13:1160966. doi: 10.3389/fonc.2023.1160966 (PMC10202170; doi:10.3389/fonc.2023.1160966)
Supplement: Supplementary file 1 [file DataSheet_1.pdf]

Patient interview guide

AML Preference Study

**TITLE**	Interview Guide: Semi-structured qualitative interviews with Acute Myeloid Leukemia patients who are not candidates for standard induction chemotherapy
**VERSION**	Version 1.0
**DATE**	18 November 2021
**CONDUCTED BY**	IQVIA
**QUOTAS**	15 interviews per country (UK, Spain and Germany)

How to use this document?

Interviews will be performed individually with each participant and are anticipated to take approximately 45 minutes. However, interviews may last about 60 minutes to allow participants to take breaks if necessary. Interviews will be performed in [UK: English; ES: Spanish; DE: German] by IQVIA's research professionals.

Interviews will be audio recorded for note-taking purposes only. Transcripts from each interview will be developed.

It is intended to be a 'guide', to help the discussion and ensure all domains in relation to the research question are covered during this discussion; therefore, text and questions are not intended to be conveyed word-for-word or verbatim, or in the order presented herein.

Notes to the interviewer are in *blue italic and* instructions in **[RED]**. These are not to be read out to the interviewee.

Probes are to be asked to the patients to deepen a domain; examples (*in italics*) are to be used only if the patient has difficulties answering the questions.

Sections, objectives and timing

Guide section	Objective	Timing
1. Introduction and disclosures	Greetings, introduce structure of the interview and housekeeping rules	5 min
2. Patient's experiences with AML (diagnosis, HMA treatments, symptoms, impacts)	To understand the patient experience with AML and its treatment, and further understand their journey from diagnosis to present	5 min
3. Patient's perspective on different modes of administration	Understand the patient's preferences for different modes of administration (oral or injectable)	5 min
4. Patient's perspective on hypothetical oral treatment (Product X)	To understand the patient's perception of an hypothetical oral treatment option compared to current alternatives	8 min
5. Patient's perception of treatment scenarios	To understand patient's perception of two hypothetical treatment scenarios and assess their preferences between the two scenarios	10 min
6. Treatment-related characteristics that drive satisfaction / preferences	To understand patient's own drivers that determine treatment satisfaction/preferences and the relative importance of treatment-related characteristics	10 min
7. Close	Gather any additional insights important to this discussion that have not been covered	2 min
**Total**		**45 min**

1. Introduction and Disclosures [5 minutes]

[Interviewer script] Thank you for taking the time to participate in this interview. To set the scene, the purpose of this discussion (approximately 45-60 minutes) is to hear about your experience of living with acute myeloid leukemia and its treatment, and to explore treatment related characteristics that might influence your preferences/satisfaction. Therefore, I will be asking you a variety of questions that will help us to cover all domains in relation to this study.

However, I first want to confirm that you have provided written consent to participate. Did you sign the form saying that you consent to participate in this study? *[interviewer note]: Pause for confirmation*

□ **Patient has provided consent**

□ **Patient has NOT provided consent** *[interviewer note]: Retrieve the information confirming that the patient has signed the consent and inform them when they signed it. If they cannot confirm that they have signed the consent form:* **[TERMINATE INTERVIEW].**

During the consent process, it was explained to you this interview will be audio-recorded. A transcript/typed up version will be created from the audio-recording, with any identifying information removed to maintain confidentiality. We will only be using the recording to remind us of the important things you said so we can represent everything accurately at the end of the study. The audio recording will be stored securely until the study is completed. Once the study is completed all audio recordings will be deleted. As a reminder, all information that can identify you personally will be kept strictly confidential. This study has been reviewed and has been given a favorable opinion by WCG IRB. This is an independent group of people, called a research ethics committee to protect your safety, rights, well-being, and dignity.

Do you agree to have the interview audio-recorded? *[Interviewer note]: Pause for confirmation / denial. If denial, explain that you cannot proceed with the interview*

□ **Patient agrees to audio recording**

□ **Patient does NOT agree to audio recording** **[TERMINATE INTERVIEW]**

To keep the interview as anonymous as possible, I am going to ask that once we begin the recording, you try to not use your name or the names of friends or family (or any clinic/facility personnel) in any of your responses during the interview. But don't worry, if you happen to mention someone's full name or any information that could identify you, we will remove it from our files to maintain your confidentiality. You won't be able to be individually identified in any of the reports that will be generated from these interviews.

In addition to recording, we may have a few listeners from our research team on what is called a listen-only line. They will not be active participants in our conversation but will help with notetaking and with the platform.

Do you give permission to have project team members listen to the call?

- ☐ **Patient agrees to there may have few listeners**
- ☐ **Patient does NOT agree that listeners attend the interview**

Now I have different questions to ask you, and the conversation should take about 45 minutes, which means that we have time for breaks if needed as we have scheduled this interview for an hour.

Do you have any questions before we begin?

[Interviewer note]: Answer questions and proceed **[START RECORDING].**

We are now recording the AML interview with [Patient ID], on [Day, Month, Year] at [Time and time zone]

The recording has started. I need to have your approval recorded so can you re-confirm that you have agreed to be recorded and have consented to participate in the study?

[Interviewer note]: Pause for confirmation / denial. If denial: **[TERMINATE INTERVIEW].**

Before we begin, please be informed that if you report an adverse event related to the sponsor's product(s), I will be obligated to report it.

2. Patient's experiences with AML (diagnosis, HMA treatments, symptoms, impacts) [5 minutes]

[Interviewer note]: To initiate a comfortable dialogue with the participants, and briefly trace their journey from diagnosis to current status

[Interviewer script]: So let's begin. To start with, I'm going to ask you a few questions to further understand your experience with AML and your journey from diagnosis to where you are now.

- First, let's start on how you normally describe your condition: How do you refer to it when you mention it to others?
  - Do you say you have AML? Or leukemia? Or something else?

[Interviewer script]: Throughout our discussion, I will be referring to acute myeloid leukemia as *<patient's term> or <AML>.*

- Please tell me, to the best of your memory, when were you officially diagnosed with AML?
  - What type of healthcare professional is involved with managing your AML now? Have you always had the same healthcare professional? How often do you visit this professional at the moment? Is this in the hospital, elsewhere, or both?
  - How long does it take to get to the hospital/treatment center from your home? Is there any burdensome aspect related to going to the hospital/treatment center?
    i. *[Interviewer notes] probes on the mode of transport: If by car then traffic and parking etc. If by public transport e.g. fear of being in proximity of people increased by patient's low immune system and risks to catch infections etc. if they are dependent on others: who is driving them and are there burdensome aspects*
- Have you ever received or are you currently receiving treatment for AML?
  - To the best of your knowledge, which treatment(s) are you currently receiving for AML, and how long have you been receiving this therapy regimen?
  - How do you take your treatment? (IV, SC or Oral) *[Interviewer notes]: If necessary, take some time to describe the different modes of administration to the participant by using the following definitions **Subcutaneously** means that a shot is given under the skin using a small needle to inject the treatment into the tissue between the skin and*

*muscle. **Intravenously** means that an infusion is given into the vein for a period of time. **Orally** means that you should take a pill (i.e. tablet or capsule).*

- How long do you stay in the hospital/treatment center to receive your treatment?
- Who monitors you before/after treatment and gives you the treatment? A nurse, a doctor or someone else? How long does the monitoring last?
- What would improve your experience of taking a treatment for AML? What is the most burdensome aspect related to this treatment?
- Could you imagine a treatment that could be a better option to manage your AML? What would it do/not do differently? If so, can you describe them?
- In terms of your experience and what you know from discussions with your health care providers/other patients/online, what is missing in AML treatment for you?

- Would you see any benefit in being treated with a different dosing regimen/ different treatment profile? Different treatment setting (e.g. home)? Please explain
- Overall, how has AML (and its treatment) impacted your life? That is, how is your life different now compared to before you had AML?
    - This could be physical, emotional, financial, or effects on your daily activities, leisure, or others
    - Have you received/are you receiving help from caregivers (i.e., close friend or family member or somebody who is the primary person who you rely on in times of need)?
        - If so, how frequently are you receiving help from them within a week or a month?

3. Patient's perspective on different modes of administration [5 minutes]

[Interviewer script] In this section, I would like to elicit your perspective on how different treatments are administered (IV, SC or Oral).

*[Interviewer notes: If necessary, take some time to describe the different modes of administration to the participant by using the following definitions: **Subcutaneously** means that a shot is given under the skin using a small needle to inject the treatment into the tissue between the skin and muscle. Usually administered in hospital, it requires you to travel to hospital and should be given*

*by a trained nurse or doctor. **Intravenously** means that an infusion is given into the vein for a period of time. Usually administered in hospital, it requires you to travel to hospital and should be given by a trained nurse or doctor. **Orally** means that you should take a pill or tablet. You would be able to take this treatment at home.]*

- In general, do you have a preference for IV, SC or oral treatment?
  - Could you describe the main reason(s) guiding your preference?
  - Do you perceive a difference between IV and SC treatments? If so, please explain
- Would you see any advantage(s)/benefit(s) in taking an AML treatment which is oral vs one which requires an injection (intravenously or subcutaneously)? Please explain why
  - Would you perceive the benefits of an oral treatment over IV to be different from the benefits of an oral treatment over SC ? Please explain
- Would you have any concerns related to an oral mode of administration? Please explain why
- Would you see any advantage(s)/benefit(s) in being treated for AML with a treatment that is injected (intravenously or subcutaneously) rather than an oral treatment? Please explain why
  - Would you have any concerns related to injectable treatments? If so, would you concerns be related to IV? SC? Both? Please explain

4. Patient's perspective on the profile of an oral treatment (Product X) [8 minutes]

[Interviewer script] In this section, I would like to elicit your perspective on a hypothetical oral treatment for AML.

Imagine you are offered an oral treatment for your AML. It is a pill. You would need to take this treatment for 5 consecutive days, then you would have 23 days without treatment, then you would take it again for 5 days, followed by 23 days with no treatment, and so on. So it would be taken for 5 consecutive days in 28-day cycles. You would need to take the pill at the same time each day and you could not eat for 2 hours before taking it, or for 2 hours after taking it. You could take this medication at home.

- Does this treatment as I have described it appeal to you? Why?

- Would you have any problems taking it in the way I described (on the day and time scheduled, i.e. for 5 consecutive days at the same time each day, at least 2 hours away from any food intake)?
- What could be the main barriers that would make it difficult for you to take it as prescribed?
    - *Probe whether the dosing regimen could be a reason for treatment non-adherence (treatment must be taken at the same time, without eating for 4 hours etc.)*

I would like you to think about this oral treatment and compare it to a SC and an IV version of the same treatment. In all cases it would be taken for 5 to 7 days with a break of 23 days before starting again; for the IV and SC this would be done in the hospital. It would take about 1-3 hours, including the injection time and the monitoring time after the injection. For the oral pill you can take it at home, and the physician may decide to reduce hospital/treatment center visits. In the case of the pill you would not be able to eat for 2 hours before and 2 hours after. There is no such restriction on the IV/SC.

- Which treatment is the most appealing to you? Why?

5. Patient's perception of treatment scenarios [10 minutes]

[Interviewer script] *First, I am going to show you scenarios about a hypothetical patient who we will call "Pat" who is undergoing an AML treatment. Pat is being offered different treatment options with varying characteristics. We want to know whether you can clearly understand the treatment characteristics, what you think of the two treatment scenarios and where your preference would go between the two treatments.*

[Interview notes] Encourage the patient to make comments as they go through the vignettes, and to point out anything that doesn't make sense to them or seems unusual. Interviewers will engage participants in discussion around their comments. If conversation dries up or doesn't flow naturally, some prompts could include:

- Was this scenario clear as you read it?
    - Were there any elements that were confusing?
    - How if at all would you change the wording in these places to make it clearer?

- Was it clear to you that this scenario is describing treatment A and B with different characteristics?
- Do you understand all the details that describe these two treatment options? If not, please specify
- Do you think you understand all the details that differentiate these two treatment options? If not, please specify
- What do you think of the two treatments described in this scenario? What do you think of Treatment A? What do you think of Treatment B?
- What are the elements that you consider most important about Treatment A? About Treatment B?
- What are the differences that matter the most to you between Treatment A and Treatment B?
- How easy/difficult would you find it to make a choice here/there?
    - Would it be an easy/difficult choice?
- Why would you make that treatment choice?

[Interviewer notes]: Particular attention should be paid to the safety profile of treatments, as presented in the vignettes by asking specific questions about the tolerance/safety

- What is your opinion regarding the side effects/toxicity profile of these treatments?
- Does the side effects/toxicity profile of these treatments influence your decision/choice? Why or why not?
- What side effects would you like to be sure to avoid? What side effects are you not comfortable with?

6. Treatment-related characteristics that drive satisfaction / preferences [10 minutes]

[Interview script] *Now I would like to discuss your perspectives on AML treatments and understand what would be the most important treatment characteristics that could influence your choice when making treatment decisions together with your doctor.*

- If you were considering starting a new treatment for your AML, what questions would you have about it? What information would you most want to know about it?
- Which other factors would drive your treatment decision/choice? Please explain

- Do you currently feel as if you have a say about treatment decisions when discussing options with your doctor? Did you discuss different options with your oncologist?

  [Interviewer notes]:
  - *If yes, then ask the following questions*
  - *If no, ask patient: If you would have been asked about your treatment preferences:*
- What would you say are the most important treatments characteristics that you would take into consideration? Why are these factors the most important?
- Are there any factors that are not so important when thinking about the best treatment for AML? Why are these factors less important?

  [Interviewer notes]: The patient might have spontaneously mentioned treatment characteristics that may be considered in their treatment preferences. However, please probe on each specific concept covered by the vignettes, one by one with the following questions:

Attributes	Questions/Probes
**Efficacy**	▪ Would you consider how well AML treatment succeeds as a factor influencing your treatment decision/choice? Why or why not? How do you define how well AML treatment succeeds (or whatever term the patient uses)?<br>○ ***Overall survival****: the average amount of time the patient is alive after treatment.*<br>○ ***Overall response rate****: chance of being cancer-free (in complete remission); the percentage of patients whose cancer shrinks or disappears after AML treatment*
**Side effects / toxicity**	▪ Would you consider side effects/undesirable symptoms* while taking AML treatments as a factor influencing your treatment decision/choice? Why or why not?<br>▪ What side effects/undesirable symptoms* that you know about would you want to make sure to avoid?
**Mode of administration**	▪ Would you consider how you take AML treatment as a factor influencing your treatment decision/choice? Why or why not?
**Impact on daily life / hospitalization**	▪ Would you consider the impact that AML treatments might have **on your daily life** as a factor influencing your treatment decision/choice? Why or why not? What specific activities of daily living?<br>▪ Would you consider frequency of hospitalization while taking the medicine as a factor influencing your treatment decision/choice? Why or why not? How often in hospital is too much?<br>▪ Would you consider the time spent at the hospital while taking the medicine **or the ability for being treated at home** as a factor influencing your treatment decision/choice? Why or why not? How much time in hospital is too much?
**Dosing regimen**	▪ Would you consider **dosing regimen** (e.g. the number of doses and when they are given) of AML treatments as a factor influencing your treatment decision/choice? Why or why not?

**Therapy duration**	▪ Would you consider **how long** AML treatments last as a factor influencing your choice? Why or why not? What is acceptable?
**Financial impact**	▪ Would you consider the **financial impact** of AML treatments you might have or the economic saving to the healthcare system as a factor influencing your choice? Why or why not?
**Other**	▪ Would you consider any other concept as important that we didn't cover that you would like to share?

* *It may be useful for the interviewer to probe on various adverse effects using patient-friendly terms:*

- ***Thrombocytopenia**: A condition in which the number of platelets is below normal, resulting in the tendency to bruise and bleed more easily – May cause patients to bruise more easily, or bleed for no reason (a nosebleed or bleeding gums from teeth brushing)*
- ***Neutropenia:** A condition in which the number of neutrophils (the most numerous type of white blood cells that helps fight infection) is below normal in the blood. It may increase patient risk for infections and cause fever or mouth sores*
- ***Febrile Neutropenia**: Fever combined with a decrease in White blood cells (neutropenia) needed to fight infection*
- ***Anemia**: A condition in which the number of Red Blood Cells (The cells that carry oxygen to the body's tissues) is below normal — May make patient feel tired, weak, or short of breath*
- ***Refractory anemia**: Anemia resistant to treatment*

[Interviewer notes]: Use your notes to collect and summarize all the treatment-related characteristics mentioned and read them back to the patient. It is ok to paraphrase if long sentences made up their answers but use the patient's words where possible. Based on the treatment-related characteristics mentioned, ask the following:

- From all the treatment characteristics you shared <interviewer to list characteristics mentioned by patient>, which are the 3 most important to you that would make you choose a treatment as preferred over another?
  - What makes <characteristic ranked first> most important?
  - Could you comment on the relative importance of the different treatment-related characteristics you have mentioned?

7. Close [2 minutes]

[Interviewer note]: Thank the interviewee for participating in the interview study / Allow for the patient to provide any additional feedback / Tell the patient how compensation will be addressed

- Do you have any additional feedback that may be important to capture related to your experience with AML or your perspectives of AML treatments that we didn't cover that you would like to share?

This is the end of the AML interview with [Patient ID], on [Day, Month, Year] at [Time and time zone]
